# In Vivo Evaluation of Thiamine Hydrochloride with Gastro-Retentive Drug Delivery in Healthy Human Volunteers Using Gamma Scintigraphy

**DOI:** 10.3390/pharmaceutics15020691

**Published:** 2023-02-17

**Authors:** Li-Ying Kam, Jia-Woei Wong, Kah-Hay Yuen

**Affiliations:** 1 School of Pharmaceutical Sciences, Universiti Sains Malaysia, Minden 11800, Penang, Malaysia; 2 School of Pharmacy, Faculty of Health & Medical Sciences, Taylor’s University, 1, Jalan Taylors, Subang Jaya 47500, Selangor, Malaysia

**Keywords:** gastro-retentive drug delivery, floating tablet, thiamine hydrochloride, gamma-scintigraphy

## Abstract

A floating tablet system containing thiamine hydrochloride, a model drug with a narrow absorption window, was evaluated. The tablet was found to have a floating lag time of less than 30 s with a sustained drug release over 12 h during in vitro dissolution studies. The gastro-retentive property of the tablet in relation to the bioavailability of thiamine was determined in healthy human volunteers using gamma scintigraphy under fasted and fed conditions. The gastro-retentive time of the floating tablet could be prolonged up to 10 h under the fed state, compared to about 1.8 h in the fasted state. The prolonged gastric retention under the fed state resulted in a 2.8-fold increase in oral bioavailability of thiamine compared to that of the fasted state. There was also a 1.4-fold increase in thiamine absorption compared to that of a conventional immediate release tablet in the fed state. In the fasted state, the extent of thiamine absorption from the floating tablet was only about 70% of that absorbed from the immediate release tablet. Thus, to achieve a better performance, such floating tablet systems should be administered under a fed condition, to prolong the gastric retention time.

## 1. Introduction

A sustained release drug delivery system offers several advantages over the immediate release dosage form, as it releases drugs at a predetermined, predictable, and controlled rate [1,2]. Nevertheless, this approach is not applicable for drugs that have a narrow absorption window, such as in the stomach or upper part of the small intestine. The extent of absorption is limited due to the rapid passage of the drug with a relatively short retention time in these regions. Thus, after passing the absorption window, there will be negligible absorption. As a consequence, a drug with a narrow absorption window will generally have limited absorption in the gastrointestinal tract [3,4,5].

A gastro-retentive drug delivery system can be particularly useful for drugs with a narrow absorption window. It is suitable for drugs that are locally active in the stomach and are unstable or insoluble in the high pH environment of the intestinal tract. Various approaches have been developed to increase the retention time in the stomach, while releasing the drug in a controlled manner. These include a high-density system [6], swelling and expandable system [7,8,9], mucoadhesive system [10,11,12], and floating system [13,14,15].

A floating drug delivery system, first described by Davis in 1968 [16], is one of the more commonly used approaches for developing a gastro-retentive drug delivery system. It is a system with a bulk density lower than that of the gastric fluid, and it is capable of floating in the gastric contents for a prolonged period of time [16,17,18,19]. In general, a density of less than 1.0 g/cm^3^ is needed for a dosage form to float and the floatability of the dosage form is usually independent of its size [20]. A floating dosage form is able to remain in the stomach for a prolonged period, because it is away from the pyloric sphincter. Thus, it is protected from the peristaltic waves of the digestive phase. On the contrary, a non-floating dosage from is usually present near the pylorus, where it is prone to gastric emptying by the propelling and retropelling waves of the digestive phase. The floating system enables the contained drug to be released slowly, at a desired and controlled rate, in the stomach. With the ability to prolong gastric retention time, such a system can improve the bioavailability of drugs with a narrow absorption window, when they are formulated into a sustained release dosage form.

Thiamine, commonly known as vitamin B1, is a water-soluble micronutrient that is necessary for the normal functioning of cells, growth, and development [21]. Thiamine is an integral component in the membrane structure and functioning, such as in the axoplasmic, mitochondrial, and synaptosomal membranes [22,23,24], and it plays a crucial role in energy metabolism. However, the oral bioavailability of thiamine has been found to be low and this could be associated with a narrow absorption window. Oral absorption of thiamine mainly occurs in its free form and takes place in the proximal part of the small intestine, with minimal absorption occurring in the stomach and colon [25,26]. Furthermore, besides passive transport, thiamine can also be absorbed via the high-affinity and low-capacity saturable thiamine transporters, which could limit the absorption of thiamine [27].

The purpose of this work was therefore to formulate a floating tablet using thiamine as a model drug and evaluate its gastro-retentive properties in relation to the bioavailability of thiamine in healthy human volunteers under both fasted and fed conditions, using gamma-scintigraphy technique. The bioavailability of thiamine from the floating system was also compared to that of a conventional immediate release reference preparation.

## 2. Materials and Methods

### 2.1. Materials

The following materials were used: model drug thiamine hydrochloride (Hubei Huazhong Pharmaceutical Co., Ltd., Shanghai, China). Hydroxypropyl methylcellulose HPMC K15M (Dow Chemical Company, MI, USA), Hydroxypropyl methylcellulose HPMC E4M (Benecel^®^, Aqualon-Hercules, Alizay, France), sodium bicarbonate (Euro Chemo-Pharma, Malaysia), microcrystalline cellulose (Avicel PH102, FMC Corporation, PA, USA), magnesium stearate (Peter Greven Asia, Malaysia), Amberlite resin (IRA-420C, 100–200 wet mesh, Sigma Chemical Co., MO, USA), and Eudragit NE 30D (Rohm GmbH, Darmstadt, Germany). Amberlite IRA-420C is a strong basic anion exchange resin in Cl^−^ form, with an exchange capacity of 3.8 meq/g. All other solvents used were either analytical or liquid chromatography grades and purchased from Merck (Darmstadt, Germany).

The reference preparation was an immediate release Dyna B1 Tablet (100 mg) manufactured by Dynapharm (M) Sdn Bhd, with a batch number of 10T0592. It was a round tablet of 10 mm in diameter containing 100 mg of thiamine hydrochloride with a tablet weight of 400 mg. It was a conventional immediate release tablet with a disintegration time of less than 15 min and dissolution profile demonstrating almost complete drug release within 30 min.

### 2.2. Preparation of the Floating Tablet

The floating tablets of thiamine hydrochloride were prepared using a direct compression method and the formulations shown in Table 1. HPMC K15M and HPMC E4M were the rate controlling polymers, while sodium bicarbonate was used as the gas generation agent. Microcrystalline cellulose was added as a bulking agent and to improve the flowability and compressibility of the powder mixture.

The powder mixture was compressed into tablets, which weighed 400 mg per tablet and contained 100 mg of thiamine hydrochloride per tablet with a diameter of 10 mm. The tablets were round, shallow convex, and scored on one face, with a thickness of approximately 5 mm. Tablet hardness was approximately 75 to 85 N.

#### 2.2.1. In Vitro Dissolution Studies

The in vitro release of thiamine from the floating tablets was investigated using a USP dissolution test apparatus II (Type PTWSIII/S12/D6-PTWS3C, Pharma Test Dissolution Tester, Hainburg, Germany) with a stirring speed of 50 rpm and a temperature of 37.0 ± 0.5 °C. The tablets were evaluated in 900 mL of three different dissolution medium, namely 0.1 M hydrochloric acid (HCl), phosphate buffer at pH 3, and phosphate buffer at pH 5. The two latter dissolution medium were employed to simulate the gastric pH in a fed condition. Approximately 5 mL of samples was withdrawn at times of 1, 2, 3, 4, 6, 8, 10, and 12 h, and the thiamine content in the samples was determined using an ultraviolet/visible (UV/VIS) spectrophotometer (D2000, Hitachi, Tokyo, Japan), with a detection wavelength of 260 nm. The dissolution studies were conducted in six replicates for each medium. In addition, the reference preparation was also subjected to dissolution study in media with pH 1.2, using the same procedures described above, with the exception that the sampling was conducted at 5, 10, 15, 20, 30, and 45 min, in view of it being an immediate release tablet.

The floating lag time and total floating time were also determined by visual observation. The former was defined as the time taken for the tablets to move from the bottom of the vessel to the surface of the medium, while the latter was the total duration of the tablets remaining constantly floating on the surface of the medium [28].

#### 2.2.2. Radiolabelling the Floating Tablet with Technetium-99 (^99m-^Tc)

Before labelling the tablets, a hole of approximately 1 mm diameter was carefully drilled through the center of the tablets using a manual hand drill. To radiolabel the tablet, around 50 mg of Amberlite resin was soaked in about 3 mL of ^99m-^Tc (Malaysia Nuclear Agency) containing about 2 GBq activity. The mixture was mixed with intermittent shaking for about 30 s and left to stand for about 15 min. This step was repeated once, and thereafter the radiolabeled Amberlite resin was washed with normal saline to remove excess free ^99m-^Tc. It was then dried and mixed with Eudragit NE 30D latex. The radiolabeled resin was then packed into the drilled hole of the tablet to obtain a radioactivity of approximately 100 MBq per tablet. The tablets were left for approximately 20 h for coalescence of the Eudragit polymer. As the half-life of ^99m-^Tc is 6 h, the radioactivity of the tablet was approximately 10 MBq (after three half-lives) at the time it was administered to the human volunteers during the conduct of the in vivo oral bioavailability study. The radioactivity of the labelled tablets was determined and confirmed using an Atomlab 200 gamma detection well (Biodex Medical System).

The radiolabeled floating tablets were subjected to dissolution studies, using procedures described in Section 2.3, to determine the effect of drilling a hole and filling it with Eudragit NE 30D latex on its dissolution characteristics.

### 2.3. In Vivo Oral Bioavailability Study

This was a randomized, single-dose, four-way crossover study conducted in 8 healthy adult male volunteers, aged from 21 to 30 years and with body mass index (BMI) of not more than 30. The volunteers were briefed about the nature of the study prior to providing their written informed consent to participate in the study. Clinical evaluations conducted during screening included detailed medical history, physical examinations, and laboratory tests, namely a liver function test, renal function test, and full blood count. Volunteers with a history or suspicion of drug dependence and/or alcohol abuse, significant clinical deviation from normal as determined by investigators, and volunteers with a history of or ongoing chronic diseases including gastrointestinal disorders were excluded. Other exclusion criteria included donation of blood in the past 8 weeks; heavy smoker; and inability to read, understand, and comply to the study protocol or to give consent. The volunteers were also required to refrain from taking any medication and vitamin supplementation one week before and during the study. The study protocol was approved by the Joint Ethics Committee on Clinical Studies of School of Pharmaceutical Sciences, USM & Hospital Lam Wah Ee, and all procedures were conducted in accordance with the Malaysian Good Clinical Practice (GCP) Guideline.

The volunteers were randomly divided into 4 groups of 2 each. There were 4 study periods, with each study period being separated by a washout period of one week. During each study period, the volunteers were administered with the study preparations in both fed and fasted states, based on the sequence shown in Table 2. In the fasted state, the tablet was administered after an overnight fast of at least 10 h, while in the fed state, the volunteers who had fasted for at least 10 h were served a standard breakfast 30 min prior to administration of the study preparations.

The radiolabeled floating tablet (test preparation) was administered with 240 mL of radiolabeled drinking water containing approximately 5 MBq activity of ^99m-^Tc, for the purpose of outlining the stomach in the volunteers. The drinking water was radiolabeled by reconstituting 4.5 mg of diethylenetriamine pentaacetic acid (DTPA) (Cis Bio International, Paris, France) with 2 mL of ^99m-^Tc containing approximately 5 MBq before making the volume up to 240 mL of drinking water. The reference preparation, which was an immediate release tablet, was administered with 240 mL of drinking water without radiolabeling. 

The volunteers were not allowed to drink water at least 1 h before dosing, except that served during breakfast and used for administration. Food was withheld for 4 h after dosing, while water was only allowed 2 h post dose. Standardized meals were served at 4 and 10 h after dosing. Volunteers were required to stay in an upright position and not in a supine position during the first 4 h after administration.

For volunteers who were dosed with test preparation, 5 mL blood samples were withdrawn at 0 (predose), 20, 40 min, 1, 1.5, 2, 3, 4, 6, 8, 10, 12, and 16 h post dose, while for those who received the reference preparation, blood samples were withdrawn at 0 (predose), 20, 40 min, 1, 1.5, 2, 3, 4, 6, 8, 10, and 12 h post dose. Plasma samples obtained from the blood samples were kept frozen at −20 °C until analysis.

Gamma scintillation was conducted using a gamma camera (Siemens Dual Head, Ecam Plus). To ensure the same anatomical position of each volunteer was taken during each image acquisition, two reference markers of 1 MBq activity each were attached to the skin of the volunteers as anatomical landmarks of the left lobe of the liver and lower right coastal margin. At the 0 time (predose), dynamic acquisition was performed for 1 min, and thereafter static images were acquired at 15-min intervals until gastric emptying of the floating tablet was completed. Acquisition was performed 60 s per frame for the first 6 h, and the capture time was increased to 120 s per frame thereafter to compensate for the radioactivity decay of ^99m-^Tc. The imaging was conducted up to 10 h post-dose and computer software (e-Soft) was used for the data collection and viewing.

#### 2.3.1. Analysis of Thiamine in Human Plasma

Thiamine plasma concentrations were analyzed using a validated liquid chromatographic tandem mass spectrometry (LCMSMS) method. The LCMSMS comprised an Agilent 1200 Series liquid chromatograph system (Agilent Technologies, Waldbronn, Germany) coupled with a Applied Biosystems API 3200 triple quadrupole mass spectrometer (Applied Biosystems/MDS Sciex, ON, Canada) with electrospray ionization. Acquisition and data analysis were performed using Analyst version 1.4.2 software. A Luna 3μ CN 100A column (100 × 2.0 mm) (Phenomenex, Torrence, CA, USA) was used for the chromatographic separation and the mobile phase consisted of 5 mM of ammonium acetate and acetonitrile at a ratio of 7:3 (*v*/*v*) with pH adjusted to 3.5 with a flow rate of 0.2 mL/min in isocratic mode. The injection volume was 20 μL. The mass analysis was conducted in positive mode, with the *m*/*z* transition for thiamine and atenolol (internal standard) at 264.6 > 121.8 and 267.1 > 144.8, respectively. The results of the analytical validation are shown in Tables S1 and S2.

Prior to analysis, the plasma samples were cleaned up using liquid–liquid extraction. The plasma samples were added to internal standard (atenolol) solution and basified using saturated potassium carbonate and thereafter extracted using dichloromethane-butanol (1:1, *v*/*v*) as the extraction solvent. The extraction solvent was subsequently isolated and re-extracted using 1% acetic acid. Thiamine in the acidic solution was then injected into the LCMSMS.

Being a food component, endogenous thiamine was inherently present in plasma, and hence endogenous (baseline) levels of thiamine were subtracted for quantification of the thiamine concentration during method validation and actual analysis of thiamine samples obtained in the in vivo oral bioavailability study. The above method was validated for its between- and within-day accuracy and precision, linearity, specificity, and extraction recovery. The stability of thiamine in the plasma samples was also determined and found to be stable for at least 2 months when stored in an amber glass bottle at −20 °C.

#### 2.3.2. Data Analysis of the In Vivo Oral Bioavailability Study

The bioavailability and pharmacokinetics of thiamine were assessed using the parameters peak plasma concentration (C_max_), time to reach peak plasma concentration (T_max_), and area under the plasma concentration versus time curve from time 0 to the time of the last measurable concentration (AUC_0-t_). Both C_max_ and T_max_ values were obtained directly from the plasma concentration data, while the AUC_0-t_ was calculated using the trapezoidal formula.

Statistical analysis of C_max_ and AUC_0-t_ was conducted using an analysis of variance (ANOVA) procedure that distinguished effects due to group, subject/group, period, and treatment [29]. The C_max_ and AUC_0-t_ values were logarithmic transformed prior to analysis. Statistically significant difference was considered at *p* < 0.05.

The images obtained from the gamma scintigraphy of volunteers dosed with floating tablets were evaluated to determine the gastric retention time, which indicated the total residence time of the tablet in the stomach. This was determined based on the time when the floating tablet left the area outlined by the radiolabeled solution in the stomach. Therefore, the gastric retention time was the time of the image that was captured preceding the first image that showed evidence of the floating tablet being emptied from the stomach.

## 3. Results

### 3.1. In Vitro Dissolution Studies

**Figure 1:**
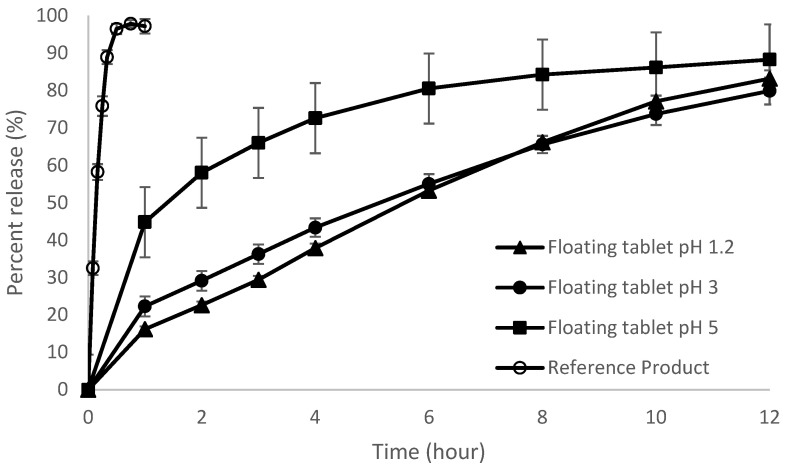
Dissolution profiles of the reference product in pH 1.2 and floating tablets in pH 1.2, 3, and 5 (*n* = 6, mean ± SD).

The in vitro release of thiamine from the floating tablets is shown in Figure 1. In dissolution medium of pH 1.2 and 3, the drug release profiles were quite similar, although not superimposable. Thiamine was gradually released from the floating tablet, with more than 80% being released within 12 h. The tablets swelled rapidly when they came into contact with aqueous medium, to form a gel barrier with the thiamine diffusing slowly out from the tablets, to attain a sustained release profile. A gradual release of thiamine instead of an initial burst effect could be achieved, as HPMC K4M and HPMC E4M are polymers with relatively low viscosity and hence they could swell rapidly to form a gel layer surrounding the tablet. In contrast, higher viscosity polymers such as HPMC K100M would have a slower swelling rate accompanied by a delay in the formation of gel barrier, and therefore they would not be able to provide effective retardation of the initial thiamine release [30]. The floating tablets in dissolution medium with pH 5 displayed a much higher percentage of thiamine release in the first hour of dissolution compared to that in dissolution mediums with pH 1.2 and 3. This could have been due to changes in the hydration properties of the gel, which were influenced by the presence of ions in the phosphate buffer medium [31]. Buffered dissolution medium has been found to increase the release of verapamil [31] and propranolol [32] from HPMC matrices. The increase in the thiamine release rate could also be attributed to a reduction in the hydrodyanamic radius of thiamine as a result of decreased polarity in higher pH conditions. The thiamine molecules carried less charges and could be transported through the narrow space between the polymer chain and diffused more rapidly out of the tablet matrix [31,33].

When the sodium bicarbonate in the floating tablet was in contact with the acidic dissolution medium, carbon dioxide was entrapped in the matrix tablet and reduced the density of the tablet, causing it to float. The floating lag times in all three pHs were very similar, with values below 30 s, while the total floating time was more than 12 h. The floating tablets could achieve rapid buoyancy, even in an elevated pH environment (pH 5), indicating that the tablet would be able to float rapidly in fed state, to escape the expulsion from the stomach by the housekeeper wave of the migrating myoelectric complex (MMC) cycle in the gastrointestinal tract. The tablets could also remain in a floating position throughout the duration of thiamine release.

**Figure 2:**
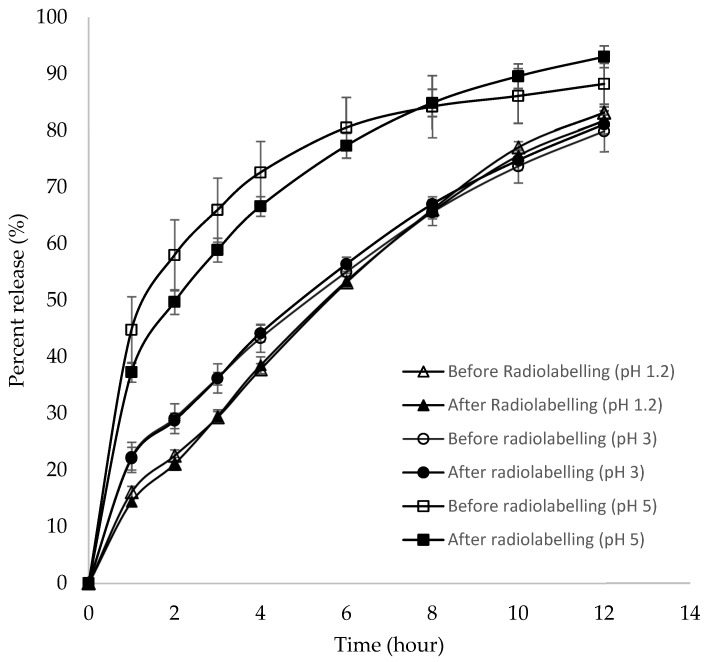
Dissolution profiles of the floating tablets before and after radiolabeling at pH 1.2, 3, and 5 (*n* = 6, mean ± SD).

The in vitro dissolution profiles of the floating tablet before and after radiolabeling (Figure 2) were essentially similar and superimposable with the respective mediums of pH 1.2 and 3. In pH 5, the release rate of the radiolabeled tablet was slightly slower than that before radiolabeling but increased thereafter. Similarity factor (f2) was calculated using a model-independent mathematical approach [34], to compare the dissolution profiles before and after radiolabeling, and the values obtained were 91.2, 92.8, and 62.4 in pH 1.2, 3, and 5, respectively. The results suggested that the drug release rates before and after radiolabeling were similar in all pH medium, indicating that the radiolabeling procedure did not affect the rate of drug release. Similarly, the floatability of the tablets after radiolabeling was not affected by the floating lag time, and the total floating time remained less than 30 s and more than 12 h, respectively.

The dissolution profile of the reference preparation showed that thiamine was completely released within 30 min, which is in line with the dissolution characteristics of an immediate release tablet.

### 3.2. In Vivo Oral Bioavailability Study

**Figure 3:**
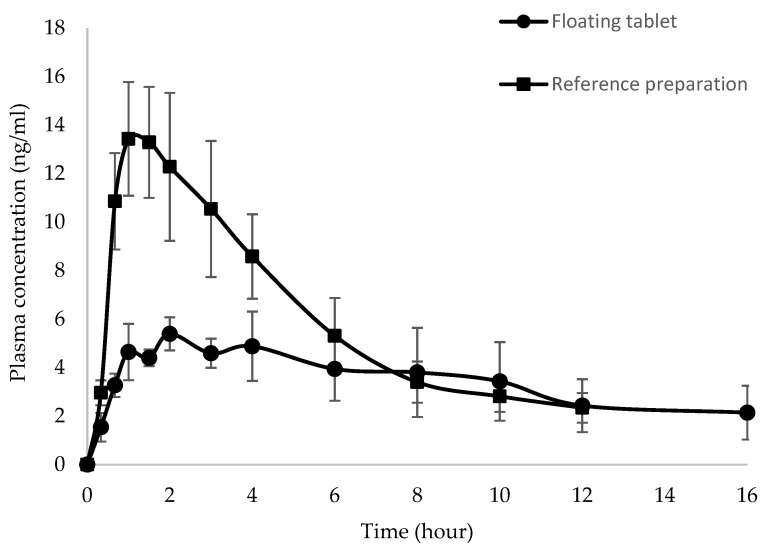
Mean plasma concentration versus time profiles of thiamine after administration of the floating tablet and reference preparation under the fasted condition (*n* = 8, mean ± SEM).

The mean plasma thiamine concentration versus time profiles of the floating tablet and reference preparation dosed in a fasted state are shown in Figure 3. The individual numerical values of C_max_, T_max_, and AUC_0-t_, and 90% confidence interval for the ratio of C_max_ and AUC_0-t_ values for floating tablet over those of the reference preparation in the fasted state are shown in Table 3. The C_max_ of the reference product was significantly higher than that of the floating tablet (*p* = 0.0189), and the 90% confidence interval for the C_max_ ratio was 0.3–0.8. These results indicated that the reference preparation had a more rapid rate of absorption, whereas the absorption of thiamine from the floating tablet was slow and sustained. This was also reflected in the mean T_max_ values, whereby the floating tablet had larger T_max_ values than the reference preparation. The AUC_0-t_ of the floating tablet was lower than the reference preparation, although the difference was not statistically significant (*p* = 0.1631). The 90% confidence interval for the AUC_0-t_ ratio was 0.3–1.1, indicating that the extent of thiamine absorption from the floating tablet was about 70% of that absorbed from the reference preparation during the first 12 h.

**Figure 4:**
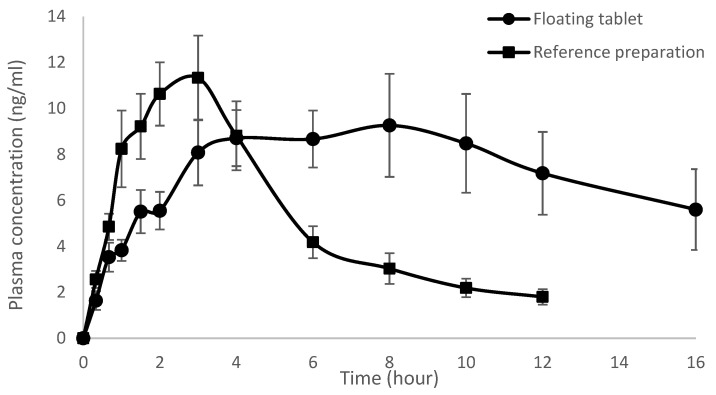
Mean plasma concentration versus time profiles of thiamine after administration of the floating tablet and reference preparation under the fed condition (*n* = 8, mean ± SEM).

The mean plasma thiamine concentration versus time profile of the floating tablet and reference product dosed under the fed condition are shown in Figure 4. The individual numerical values of C_max_, T_max_, and AUC_0-t_, and 90% confidence interval for the ratio of C_max_ and AUC_0-t_ values for the floating tablet over those of the reference preparation in the fed state are shown in Table 4. The C_max_ values of the floating tablet and reference preparation were almost comparable, and no statistically significant difference was observed between the two preparations (*p* = 0.5365). The T_max_ values obtained from the floating tablet were significantly higher than those of the reference preparation, which could be attributed to a slower release rate of thiamine and a prolonged gastric retention time of the floating tablet, resulting in a prolonged absorption period. The AUC_0-t_ of the floating tablet was also significantly higher (*p* = 0.0329) than that of the reference preparation, and the 90% confidence interval for the AUC_0-t_ ratio was 1.2–1.7, suggesting an approximately 1.4-fold increase in the extent of thiamine absorption from the floating tablet compared to the reference preparation.

A comparison was also made between the bioavailability of thiamine from the floating tablet dosed under the fasted and fed states, and it was observed that the absorption of thiamine was markedly increased when it was dosed in the fed state. The C_max_ (*p* = 0.0016) and AUC_0-t_ (*p* = 0.0019) values in the fed state were significantly higher than those in the fasted state. The 90% confidence interval in the fed state over the fasted state was 1.2–1.8 for the C_max_ ratio and 1.5–4.2 for the AUC_0-t_ ratio, indicating that the peak plasma concentration had increased by about 1.5-fold, while the absorption was increased by about 2.8-fold when the floating tablet was administered in the fed condition compared to administration in the fasted state. In addition, the T_max_ values of each volunteer were all significantly higher than those of the fasted state.

### 3.3. In Vivo Evaluation of the Floating Properties Using Gamma Scintigraphy

**Figure 5:**
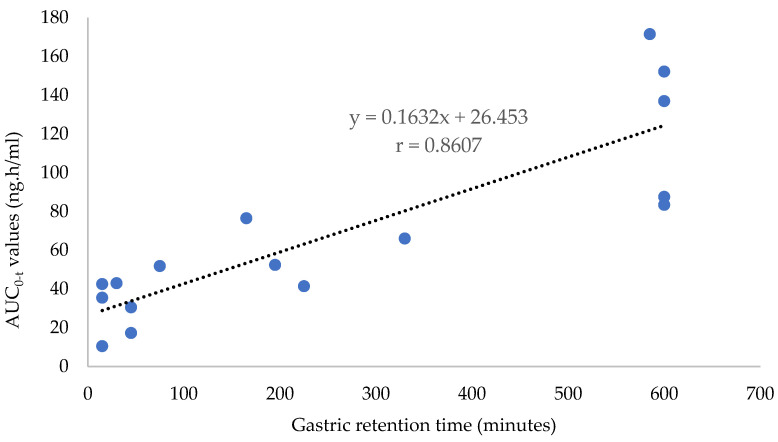
Gastric retention times obtained in fasted and fed state versus the corresponding AUC_0-t_ values of the floating tablets.

The gastric retention times of the floating tablet in the fasted and fed states are shown in Table 5. Sample images of gamma scintigraphy for a volunteer in the fasted and fed state at different time intervals after oral administration of floating tablet are presented in Figures S1 and S2, respectively. The gastric retention time in the fasting state ranged from 15–75 min, being relatively short in comparison with other reported studies, except for volunteer 2, who retained the floating tablet for more than 600 min after dosing. Whitehead et al. [35] reported the onset of gastric emptying was 32–102 min for floating beads, while Sato et al. [36] reported that microballoons could be retained in the stomach for 60–90 min under a fasted state. Bomma et al. [37] reported a much longer gastric residence time of 180 min for norfloxacin floating tablets in a fasted state and an even longer residence time of 225 min for cefuroxime axetil floating tablets [38]. On the other hand, in the fed state, the tablets were retained in the stomach for at least 165 min, with a few volunteers (volunteers 4, 6, and 8) retaining the tablets in the stomach for more than 600 min. The longer gastric residence time in the fed state was reflected in the higher oral bioavailability of thiamine compared to the fasted state. When the gastric retention times obtained in the fasted and fed state were plotted against the corresponding AUC_0-t_ values of the floating tablets, a Pearson correlation coefficient (r) of 0.8607 was obtained, indicating a positive correlation between the two parameters (Figure 5). The floating tablets in the present fed study were retained longer in the fed state compared to those reported in other studies. Strusi et al. [39] reported that a Dome Matrix^®^ void configuration had a gastric retention time of 145–275 min in the fed state, while Sato et al. [36] reported that their floating dosage forms were buoyant in the gastric contents for up to 300 min.

## 4. Discussion

Generally, feeding state of the stomach has a significant influence on the gastric retention time of a floating tablet, which in turn will affect the bioavailability of thiamine. In a fasted state, the floating tablet may not float well due to an insufficient volume of fluid in the stomach. Moreover, the floating tablet had a high probability of being emptied rapidly from the stomach by the housekeeper wave and passed through the absorption window in the proximal part of the small intestine, when the thiamine was still being released in a sustained manner. Minimal or no absorption took place, even though the release of drug was continuous along the distal part of the small intestine, and this consequently compromised the bioavailability of the thiamine. In contrast, the reference preparation disintegrated completely, to release the entire amount of thiamine for absorption and hence a faster and higher amount of thiamine could be absorbed when it passed the proximal part of the small intestine. Similar findings were reported by Groning et al. [40], where a gastro-retentive collagen tablet was administered to the volunteers under a fasted condition. The collagen tablet containing riboflavin was not retained for a prolonged period and led to reduced renal excretion of riboflavin.

In contrast, the increase in the extent of absorption in the presence of food could be attributed to the delayed onset of gastric emptying, which allowed the floating tablet to remain in the stomach for a prolonged period, to gradually release thiamine from the floating tablet, and hence there was an increase in the absorption in the small intestine. Sawicki [41] reported similar findings, whereby the AUC of verapamil in a floating dosage form was increased by about 60% compared to that of an immediate release tablet, and a lower C_max_ value was obtained with the floating tablet.

The larger T_max_ values of each volunteer observed under the fed state were also consistent with those of dissolution studies, wherein a faster rate of thiamine release was evident at pH 5 that represented the fed state compared to the pH 1.2 of the fasted state.

In a fasted state, the housekeeper wave of the MMC cycle is responsible for sweeping large, indigestible materials out of the stomach every 80 to 120 min [38,42]. Therefore, the floating tablet administered just before or during the occurrence of housekeeper wave could be emptied rapidly from the stomach. In contrast, a floating tablet would have a prolonged period of gastric retention if it was administered right after the housekeeper wave, as it would only be cleared by the next housekeeper wave. This explained for the variable gastric retention time of the floating tablets administered in a fasted state observed in the present study [43]. Moreover, the volume of liquid in the stomach may not be sufficient to swell and float the tablet, and hence the entire stomach contents could have been emptied into the small intestine by the housekeeper wave. In the fed state, the presence of food in the stomach delayed the onset of gastric emptying and also decreased the gastric emptying rate, resulting in a delayed evacuation of the dosage form from the stomach.

## 5. Conclusions

The presence of food had a profound effect on the gastric emptying and hence gastric retention time of the floating tablet. Gastric emptying was delayed in the fed state and the floating tablet was thus retained in the stomach for a prolonged period, allowing thiamine to dissolve in a controlled manner and be available for continuous absorption at the proximal part of the small intestine, hence leading to a better bioavailability. The overall bioavailability for thiamine was thus increased when the floating tablet was dosed in the fed state. However, under the fasted condition, the floating tablet did not appear to prolong the gastric emptying time, resulting in a lower bioavailability. Hence, the floating system should preferably be administered under a fed condition, to prolong the gastric retention time and attain a better bioavailability.

## Figures and Tables

**Table 1:** Formulation of the thiamine hydrochloride floating tablet.

Ingredients	Weight per Tablet (mg)	Percentage by Weight (%)
Thiamine hydrochloride	100	25.0
HPMC K15M	108	27.0
HPMC E4M	72	18.0
Sodium bicarbonate	100	25.0
Microcrystalline cellulose PH102	16	4.0
Magnesium stearate	4	1.0
Total tablet weight	400	

**Table 2:** Sequence of administration of floating tablet and reference tablet under fasted and fed conditions.

Group	Sequence of Administration			
	Period 1	Period 2	Period 3	Period 4
1	T (Fasted)	T (Fed)	R (Fasted)	R (Fed)
2	T (Fed)	T (Fasted)	R (Fed)	R (Fasted)
3	R (Fasted)	R (Fed)	T (Fasted)	T (Fed)
4	R (Fed)	R (Fasted)	T (Fed)	T (Fasted)

T = Test preparation containing radiolabeled thiamine hydrochloride 100 mg floating tablet administered with radiolabeled drinking water, R = Reference preparation containing non-radiolabeled immediate release thiamine hydrochloride 100 mg tablet administered with unlabeled mineral water.

**Table 3:** Individual numerical values of C_max_, T_max_, and AUC_0-t_, and 90% confidence interval for thiamine after administration of floating tablet and reference preparation in the fasted state.

Volunteer	Floating Tablet			Reference Preparation		
	C_max_ (ng/mL)	T_max_ (h)	AUC_0-t_ (ng.h/mL)	C_max_ (ng/mL)	T_max_ (h)	AUC_0-t_ (ng.h/mL)
1	4.4	3.0	35.5	7.2	1.5	34.3
2	17.3	8.0	136.9	19.9	1.5	132.6
3	6.3	4.0	42.5	30.7	2.0	146.8
4	7.2	3.0	51.8	11.8	2.0	75.6
5	6.5	2.0	30.5	22.0	1.0	59.0
6	13.1	1.0	43.0	11.3	1.0	15.7
7	4.9	1.5	10.5	10.3	1.0	43.5
8	5.5	1.5	17.3	18.9	3.0	89.6
Mean	8.2 *	3.0	46.0	16.5	1.6	74.6
SD	4.6	2.3	39.2	7.8	0.7	46.5
C.I.	0.3–0.8		0.3–1.1			

* *p* < 0.05 when compared with that of reference preparation, SD is standard deviation, C.I. is the 90% confidence interval of the values obtained with the floating tablet over those of reference preparation.

**Table 4:** Individual numerical values of C_max_, T_max_, and AUC_0-t_, and 90% confidence interval for thiamine after administration of the floating tablet and reference preparation in the fed state.

Volunteer	Floating Tablet			Reference Preparation		
	C_max_ (ng/mL)	T_max_ (h)	AUC_0-t_ (ng.h/mL)	C_max_ (ng/mL)	T_max_ (h)	AUC_0-t_ (ng.h/mL)
1	8.4	6.0	66.0	8.6	3.0	47.1
2	20.6	8.0	171.4	19.4	1.0	101.2
3	13.2	4.0	76.5	17.4	2.0	78.4
4	16.5	8.0	152.1	23.5	3.0	104.5
5	4.8	3.0	41.4	10.4	2.0	52.3
6	11.1	8.0	87.5	10.8	3.0	51.3
7	8.2	4.0	52.4	6.3	3.0	21.7
8	11.4	8.0	83.4	8.0	1.5	51.4
Mean	11.8	6.1	91.4 *	13.0	2.3	63.5
SD	5.0	2.2	46.4	6.2	0.8	28.7
C.I.	0.7–1.1		1.2–1.7			

* *p* < 0.05 when compared with that of reference preparation, SD is standard deviation, C.I. is the 90% confidence interval of the values obtained with the floating tablet over that of reference preparation.

**Table 5:** Gastric retention time for thiamine floating tablets in healthy volunteers in fasted and fed states.

Volunteer	Fasted (Minutes)	Fed (Minutes)
1	15	330
2	More than 600	585
3	15	165
4	75	More than 600
5	45	225
6	30	More than 600
7	15	195
8	45	More than 600

## Data Availability

The data supporting the findings of this study are available on request from the corresponding author upon reasonable request.

## Supplementary Materials

The following supporting information can be downloaded at: https://www.mdpi.com/article/10.3390/pharmaceutics15020691/s1. Figure S1: Gamma scintigraphy images of floating tablet for volunteer 8 in fasted state at (a) 45 min prior to gastric emptying of the tablet and (b) 60 min after gastric emptying of the tablet. Figure S2: Gamma scintigraphy images of floating tablet for volunteer 8 in fed state at (a) 60 min prior to gastric emptying of the tablet and (b) 600 min prior to gastric emptying of the tablet. Table S1: Specificity, linear range, limit of detection and limit of quantification for analysis of thiamine. Table S2: Extraction recovery, within-day and between-day precision and accuracy values (n = 6) for thiamine.
