# Supplementary material for: In Vivo Evaluation of Thiamine Hydrochloride with Gastro-Retentive Drug Delivery in Healthy Human Volunteers Using Gamma Scintigraphy
Source: Pharmaceutics. 2023 Feb 17;15(2):691. doi: 10.3390/pharmaceutics15020691 (PMC9960539; doi:10.3390/pharmaceutics15020691)
Supplement: Supplementary file 1 [file pharmaceutics-15-00691-s001.zip › Figure S2.pdf]

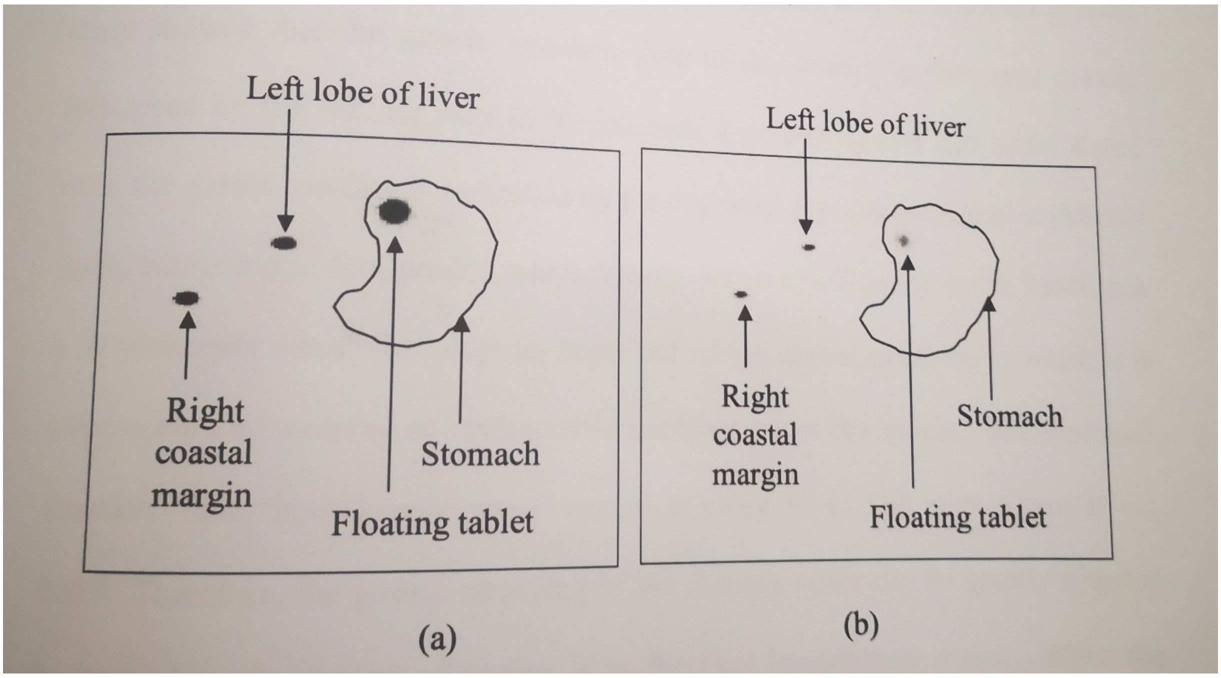

Figure S2: Gamma scintigraphy images of floating tablet for volunteer 8 in fed state at (a) 60 minutes prior to gastric emptying of the tablet and (b) 600 minutes prior to gastric emptying of the tablet.
