# Supplementary material for: In Vivo Evaluation of Thiamine Hydrochloride with Gastro-Retentive Drug Delivery in Healthy Human Volunteers Using Gamma Scintigraphy
Source: Pharmaceutics. 2023 Feb 17;15(2):691. doi: 10.3390/pharmaceutics15020691 (PMC9960539; doi:10.3390/pharmaceutics15020691)
Supplement: Supplementary file 1 [file pharmaceutics-15-00691-s001.zip › Table S1.pdf]

Table S1: Specificity, linear range, limit of detection and limit of quantification for analysis of thiamine

Validation Parameters	Results
Specificity	Thiamine is an endogenous compound and hence thiamine peak could be observed in the blank plasma. In order to quantify the actual spiked thiamine and absorbed thiamine in the method validation samples and pharmacokinetic samples respectively, it was necessary to subtract the endogenous level of thiamine.<br><br>For the internal standard (atenolol), no interference was observed at its retention time in the blank plasma.
Linear Range	0.625 ng/ml – 80.0 ng/ml, r = 0.9995
Limit of Detection	0.313 ng/ml
Limit of Quantification	0.625 ng/ml
