# Supplementary material for: In Vivo Evaluation of Thiamine Hydrochloride with Gastro-Retentive Drug Delivery in Healthy Human Volunteers Using Gamma Scintigraphy
Source: Pharmaceutics. 2023 Feb 17;15(2):691. doi: 10.3390/pharmaceutics15020691 (PMC9960539; doi:10.3390/pharmaceutics15020691)
Supplement: Supplementary file 1 [file pharmaceutics-15-00691-s001.zip › Table S2.pdf]

Table S2: Extraction recovery, within-day and between-day precision and accuracy values (n=6) for thiamine

Concentration (ng/ml)	Extraction Recovery[1]		Within-Day		Between-Day	
	Mean (%)	C.V. (%)	Precision[2] (%)	Accuracy[3] (%)	Precision (%)	Accuracy (%)
0.625	78.1	10.7	12.8	94.3	8.2	105.5
1.25	70.9	12.1	8.5	107.8	8.8	94.8
10.0	70.8	8.3	12.2	98.4	5.6	102.5
80.0	63.8	10.5	8.1	91.7	7.2	96.9

[1] Extraction recovery was calculated by comparing the response of the thiamine obtained after extraction with that of the corresponding concentration of thiamine working solution in 1% acetic acid (without extraction). Extraction recovery for atenolol (internal standard) was approximately 50%.

[2] Precision was denoted using coefficient of variation.

[3] Accuracy was expressed as percentage of the true value.
